# BMP signaling and cellular dynamics during regeneration of airway epithelium from basal progenitors

**DOI:** 10.1242/dev.126656

**Published:** 2016-03-01

**Authors:** Tomomi Tadokoro, Xia Gao, Charles C. Hong, Danielle Hotten, Brigid L. M. Hogan

**Affiliations:** 1 Department of Cell Biology, Duke Medicine, Durham, NC 27710, USA; 2 Department of Medicine-Cardiovascular Medicine, Vanderbilt Institute of Chemical Biology, Vanderbilt University School of Medicine, Nashville, TN 37212, USA; 3 Department of Medicine, Division of Cardiology, Duke Medicine, Durham, NC 27710, USA

**Keywords:** Airway epithelium, Basal cells, Cell shedding, Apoptosis, BMP signaling, SO_2_ injury, Regeneration, Homeostasis

## Abstract

The pseudostratified epithelium of the lung contains ciliated and secretory luminal cells and basal stem/progenitor cells. To identify signals controlling basal cell behavior we screened factors that alter their self-renewal and differentiation in a clonal organoid (tracheosphere) assay. This revealed that inhibitors of the canonical BMP signaling pathway promote proliferation but do not affect lineage choice, whereas exogenous Bmp4 inhibits proliferation and differentiation. We therefore followed changes in BMP pathway components *in vivo* in the mouse trachea during epithelial regeneration from basal cells after injury. The findings suggest that BMP signaling normally constrains proliferation at steady state and this brake is released transiently during repair by the upregulation of endogenous BMP antagonists. Early in repair, the packing of epithelial cells along the basal lamina increases, but density is later restored by active extrusion of apoptotic cells. Systemic administration of the BMP antagonist LDN-193189 during repair initially increases epithelial cell number but, following the shedding phase, normal density is restored. Taken together, these results reveal crucial roles for both BMP signaling and cell shedding in homeostasis of the respiratory epithelium.

## Abstract (precis)

**Summary:** In the mouse airway epithelium, regeneration after injury involves transient downregulation of BMP signaling to promote proliferation, followed by cell shedding to restore cell density.

## INTRODUCTION

Lung function is highly dependent on the cellular composition and integrity of the mucociliary epithelium lining the airway tubes and its interactions with the underlying mesenchyme. Epithelial integrity is important because the apical junctional complexes between the luminal cells provide the first line of defense against pathogens and inhaled particles, while allowing selective paracellular transport of ions and macromolecules (Flynn et al., 2009; Macara et al., 2014; Paul et al., 2014; Rezaee and Georas, 2014). Cellular composition is important because the specialized secretory and ciliated cells produce mucus and antimicrobial agents and remove entrapped particles from the lung. Airway epithelial cells can also produce immune modulators, a function shared with the monocyte-derived cells intercalated within the surface layer (Hallstrand et al., 2014; Hammad and Lambrecht, 2008; Persson et al., 2014). Given these multiple functions it is crucial that the cellular composition and architecture of the airway epithelium are quickly repaired after damage caused by viral or bacterial infection or by inhalation of smoke or toxic gases (reviewed by Hogan et al., 2014). Indeed, there is experimental evidence that failure of epithelial repair can lead to dysregulation of the underlying stroma and fibrosis and bronchiolitis obliterans-like conditions (O'Koren et al., 2013).

The task of maintaining and repairing pseudostratified airway epithelium falls mainly on the Trp63^+^ Krt5^+^ basal cells (BCs). These lie close to the basal lamina and make up 20-30% of the total population (reviewed by Rock et al., 2010). *In vivo* lineage-tracing studies in the pseudostratified mucociliary epithelium of the neonatal and adult mouse trachea have shown that BCs can function as classical stem cells and both self-renew and give rise to ciliated and secretory cells. Notch signaling promotes this differentiation, with low levels favoring the production of ciliated cells and high levels promoting secretory cell fate (Pardo-Saganta et al., 2015b; Paul et al., 2014; Rock et al., 2011b, 2009). Recent studies indicate that the Krt5^+^ BC population is heterogeneous. Some BCs appear to function as classic multipotent stem cells, while others are thought to be progenitors already committed to a ciliated or secretory fate (Mori et al., 2015; Pardo-Saganta et al., 2015a; Watson et al., 2015).

One approach to identifying the mechanisms regulating repair of the airway epithelium is to study regeneration of the mucociliary epithelium of the mouse trachea after killing the luminal cells by brief exposure to SO_2_ gas (Borthwick et al., 2001; Gao et al., 2015; Kim et al., 2012; Pardo-Saganta et al., 2015a; Rawlins et al., 2007; Rock et al., 2011b). Following sloughing of the dead cells the BCs quickly spread to cover the denuded basal lamina, establish intercellular junctional complexes and proliferate to generate a population of progenitor cells. These differentiate into mature ciliated and secretory cells, regenerating the epithelium by ∼2 weeks after injury. Epithelial damage also triggers changes in the underlying mesenchymal layer, including an early influx of neutrophils and macrophages (Tadokoro et al., 2014).

Based on what is known about repair mechanisms in other tissues (Chen et al., 2015; Eming et al., 2014; Hsu et al., 2014; Lee and Miura, 2014; Miyoshi et al., 2012) it is likely that multiple signaling pathways work together in the epithelial and mesenchymal compartments to orchestrate regeneration of the mucociliary epithelium. To identify potential regulators of repair we have previously used a 3D organoid (‘tracheosphere') assay to screen for factors and small molecules that modulate the proliferation and differentiation of BCs and their progeny. This led to the finding that the cytokine IL6, made predominantly by Pdgfra^+^ fibroblasts in the stroma early during repair, enhances the differentiation of BCs into multiciliated cells (Tadokoro et al., 2014). Here, using the same assay, we report that inhibitors of the BMP signaling pathway function as positive regulators of BC proliferation. By contrast, exogenous BMP ligands act as inhibitors, as reported recently for human nasal epithelial cells (Cibois et al., 2015). Gene expression studies support the idea that BMP signaling between the mesenchyme and epithelium plays a role in regulating epithelial proliferation *in vivo*. We therefore tested the hypothesis that inhibiting BMP signaling by systemic administration of LDN-193189, a small-molecule BMP signaling antagonist, would enhance repair by promoting BC proliferation. LDN-193189 treatment did indeed increase the size of BC clones and the number of differentiating progenitors that accumulate during the early phase. However, we found that apoptosis and active cell extrusion subsequently restore the original cell density of the epithelial cell layer, both during normal repair and after inhibitor treatment.

## RESULTS

### BMP ligands and inhibitors regulate the formation of 3D tracheospheres from BCs

**Fig. 1.**
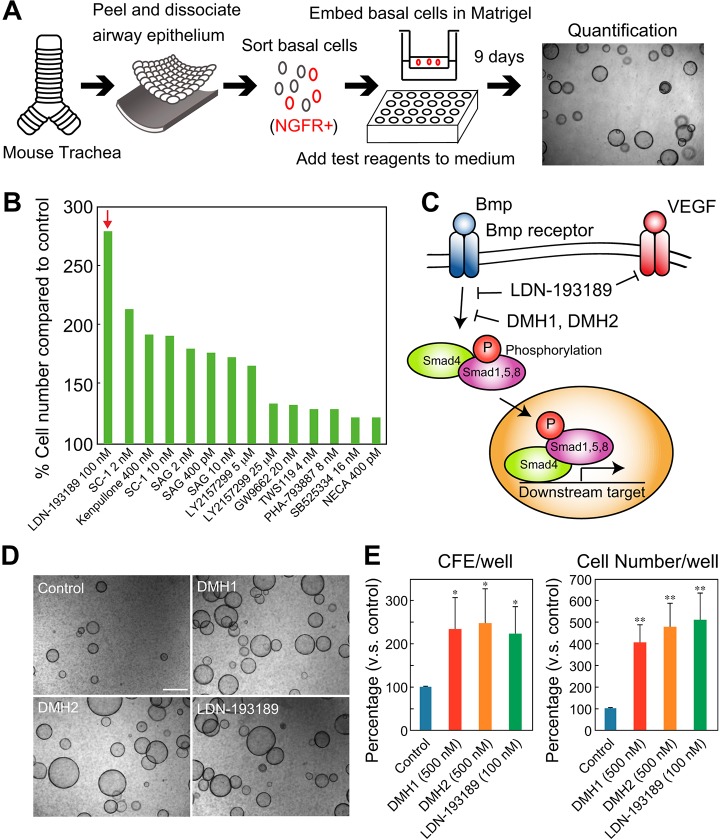
**BMP inhibitors promote cell proliferation of tracheal basal cells.** (A) Assay schematic. Ngfr^+^ basal cells (BCs) were cultured with test compounds in 50% Matrigel in 24-well inserts. The numbers of spheres and cells were quantified at day 9. To the right is a representative bright-field image of spheres at day 9. (B) The effect of potentiators/inhibitors of different signaling pathways in the assay. Bars show cell number as a percentage of the control. Data are the mean of duplicates. The red arrow indicates highest response. (C) Schematic of BMP signaling and inhibitors. LDN-193189 inhibits both BMP and VEGF signaling, whereas DMH1 and DMH2 show little or no effect on VEGF signaling. (D) Bright-field images of spheres in cultures with BMP inhibitors LDN-193189, DMH1 and DMH2. (E) The effect of BMP inhibitors on colony forming efficiency (CFE) and cell number. *P<0.01, **P<0.003 versus control (*n*=3). Scale bar: 500 µm.

To explore the signaling pathways that stimulate regeneration of airway progenitors we exploited a BC 3D organoid (tracheosphere) culture system (Fig. 1A) (Rock et al., 2009; Tadokoro et al., 2014). In this assay, single Trp63^+^ Ngfr^+^ Krt5^+^ BCs are seeded into extracellular matrix and cultured for 14 days under conditions in which they can self-renew and differentiate into either ciliated or secretory cells. At day 9, all spheres >50 μm in diameter are counted to give colony forming efficiency (CFE) and then the cultures are dissociated to estimate total cell number (proliferation). In some experiments spheres are also sectioned and examined histologically.

Fig. 1 shows the results of a screen using small compounds that are either agonists or antagonists for specific intercellular signaling pathways (Table S1). We found that LDN-193189, a derivative of dorsomorphin (DMH1), is the most effective at promoting proliferation (Fig. 1B). Since LDN-193189 inhibits BMP, VEGF and p38 (Mapk1) signaling pathways, we also tested the effect of DMH1 and DMH2 (Boergermann et al., 2010; Hao et al., 2010). These compounds more specifically block the BMP pathway by inhibiting the phosphorylation of Smads but do not inhibit non-canonical p38 kinase or VEGF signaling (Fig. 1C) (Hao et al., 2010). Both DMH1 and DMH2 promote CFE and total cell number (Fig. 1D,E), supporting a model in which BMP signaling inhibits the proliferation of tracheal BCs and their progeny through phosphorylation of Smad1/5/8. DMH1 also promoted the serial propagation of BCs in the clonal organoid culture assay (Fig. S1).

**Fig. 2.**
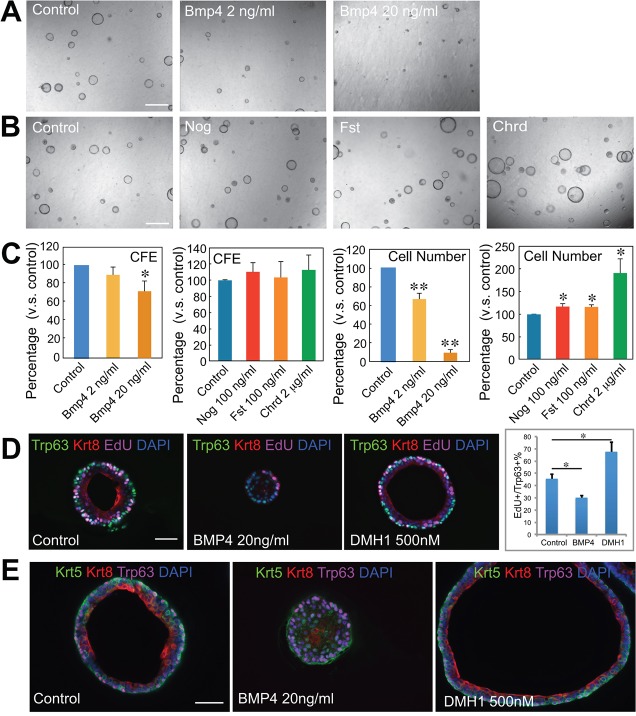
**BMP signaling regulates proliferation of tracheal BCs.** (A,B) Bright-field images of spheres treated for 9 days with (A) different concentrations of Bmp4 and (B) the BMP antagonists Nog, Fst and Chrd. (C) Effect of Bmp4 and BMP antagonists on CFE (left) and cell number (right). *P<0.01, **P<0.001 versus control (*n*=3). (D) Sections of spheres cultured for 7 days under different conditions and exposed to EdU for 2 h before harvest stained with antibodies to Trp63, Krt8 and EdU. The bar chart shows the percentage of Trp63^+^ cells that are also EdU^+^. *P<0.05. (E) Sections of spheres cultured for 9 days under different conditions stained with antibodies to Krt5, Krt8 and Trp63. Scale bars: 500 µm in A,B; 50 µm in D,E.

We next tested the effect in the sphere assay of recombinant BMP ligands that signal through Smad1/5/8 (Brazil et al., 2015; Mueller and Nickel, 2012). Bmp4 reduces CFE and total cell number in a dose-dependent manner (Fig. 2A,C). Interestingly, Bmp5, which belongs to a different subclass of BMP ligands (Bmp5, 6 and 7) than Bmp2 and Bmp4, did not have an effect when tested at the same concentrations (Fig. S2). We then tested the activities of three protein inhibitors of BMP signaling, namely noggin (Nog), follistatin 288 (Fst) and chordin (Chrd), that function by blocking the binding of ligand to cell surface receptors (Brazil et al., 2015; Iemura et al., 1998). These all significantly promoted cell number but not CFE, with the most effective being Chrd (Fig. 2B,C).

To determine the effect of Bmp4 and DMH1 on BC proliferation and differentiation we exposed spheres that had been cultured for 7 days to EdU for 2.5 h before harvesting and then fixed and analyzed them by immunohistochemistry. Both control and DMH1 spheres contained Trp63^+^ basal and Krt8^+^ luminal cells (Fig. 2D). By contrast, Bmp4-treated spheres contained only Trp63^+^ BCs. Analysis and quantification of EdU incorporation showed that the proliferation of Trp63^+^ cells was higher in DMH1-treated and lower in Bmp4-treated spheres than in controls. After 9 days of culture (Fig. 1E), Bmp4-treated spheres were still mainly composed of Trp63^+^ BCs, with very few Krt8^+^ cells around a small central lumen. By contrast, control and DMH1-treated spheres both had prominent lumens associated with Krt8^+^ luminal cells.

To test whether the effect of BMP is reversible, we switched cultures that had been exposed to 20 ng/ml Bmp4 for 7 days to either control medium or medium containing 500 nM DMH1 and continued culture for a further 7 days. Before switching, the average diameter of Bmp4-treated spheres was 51.7±2.8 µm (*n*=3). After 7 days in control medium the average diameter had increased to 120.4±1.9 µm, and to 131.1±2.9 µm in the presence of DMH1. Taken together, these studies show that Bmp4 inhibits the proliferation and differentiation of Trp63^+^ BCs but this effect can be reversed.

In a previous study, BCs from *Foxj1-GFP* transgenic mice were used to follow their differentiation into ciliated cells in organoid cultures (Tadokoro et al., 2014). Analysis of such cultures showed that LDN-193189 initially promoted the appearance of ciliated cells, but by day 14 there was no significant difference in the proportion of ciliated cells in treated cultures compared with controls (Fig. S3A). In addition, spheres exposed to LDN-193189 contained Scgb3a2^+^ secretory cells in about the same proportion as controls (Fig. S3B). Taken together with the data in Figs 1 and 2, these results suggest that inhibition of BMP signaling promotes the proliferation of BCs and their differentiation but does not, over the long-term, influence lineage choice.

### Dynamic expression of BMP signaling pathway components during repair

**Fig. 3.**
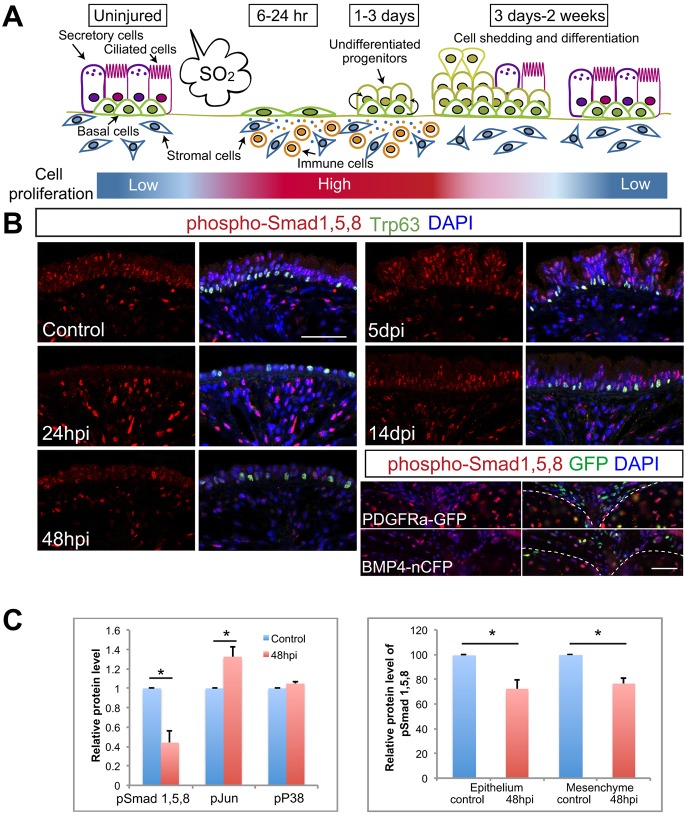
**Dynamic changes in BMP signaling during tissue regeneration.** (A) Schematic of repair of tracheal epithelium after SO_2_ injury. Luminal cells are sloughed off during the first 6-12 h after SO_2_ exposure (hpi) and BCs spread to cover the denuded area by 24 hpi. BCs proliferate and generate Krt8^+^ suprabasal descendants that accumulate and become multilayered during the first 6 days. Some differentiated ciliated and secretory cells are first detected around day 3 and regeneration of the epithelium is complete by 2 weeks. Evidence is presented here for cell shedding to restore homeostasis. (B) Phospho-Smad1/5/8 (red) levels in DAPI-stained nuclei of epithelium and mesenchyme during repair after SO_2_ inhalation. Phospho-Smad1/5/8 is seen in both Trp63^+^ BCs (green) and luminal cells. Note that not all cells are positive for phospho-Smad1/5/8. Bottom right panels show phospho-Smad1/5/8 in the intercartilage mesenchyme of uninjured tracheas. The cartilage is outlined (dashed line). Some phospho-Smad1/5/8^+^ cells are also positive for Pdgfra or Bmp4 (green). Scale bars: 50 µm. (C) Quantification of western blot analysis (Fig. S3) of phospho-Smad1/5/8, phospho-Jun and phospho-p38 in the total trachea before (control) and 48 h after (left) injury and phospho-Smad1/5/8 in the epithelium versus mesenchyme before and 48 h after injury (right). Values are the mean of triplicate (left) or duplicate (right) samples. *P<0.05.

Given our findings in *in vitro* culture, we examined the expression of a number of key components of the BMP pathway in the trachea at steady state and during repair after SO_2_ exposure. Both Ngfr^+^ basal and Ngfr^–^ epithelial cells and mesenchyme express transcripts for *Bmpr1a*, *Bmpr1b* and *Acvr1* receptors at steady state (Fig. S4A). In addition, immunohistochemistry for phosphorylated Smad1/5/8 (Fig. 3B) showed that BMP signaling is active in both basal and luminal epithelial cells at steady state. Some positive cells are also present in the intercartilage mesenchyme. This includes fibroblast-like cells that express *Pdgfra* or *Bmp4*, as judged by the nuclear expression of GFP under the control of the respective genomic loci (Fig. 3B).

By 24 h post injury (hpi), a time when more than 70% of the cells are proliferating, as judged by BrdU labeling (Rawlins et al., 2007), levels of phospho-Smad1/5/8 in the epithelium are significantly reduced. Levels remain low throughout the first 4 days of repair but, by 2 weeks, when the epithelium is fully regenerated, levels are back to normal. These findings were confirmed by western blot analysis of protein from either total tracheas or from epithelium and mesenchyme separately both before and 48 h after injury (Fig. 3C, Fig. S4B). By contrast, in total trachea, levels of phospho-p38 do not change and levels of phospho-Jun increase.

**Fig. 4.**
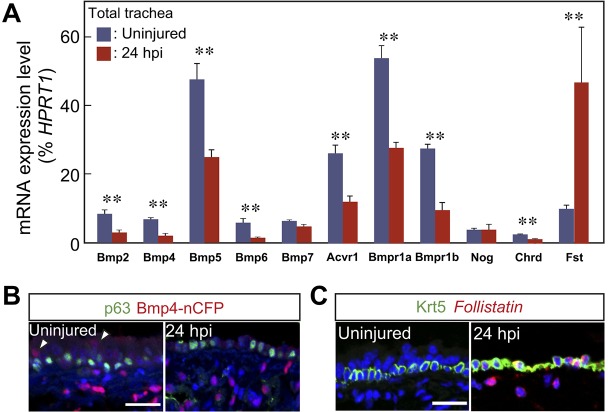
**Expression of Bmp-related genes during tissue regeneration.** (A) Quantitative RT-PCR analysis of transcripts for genes encoding some BMP ligands, receptors and antagonists in total trachea before and after injury. (B) *Bmp4-nCFP* expression (red) in trachea before and after injury. Arrowhead indicates weak expression of *Bmp4* in the epithelium above Trp63^+^ BCs (green). (C) After injury, *Fst* transcripts (red) are detected by *in situ* hybridization in both the Krt5^+^ BCs (green) and mesenchyme. **P<0.01 versus uninjured (*n*=3). Scale bars: 20 μm.

We next asked about changes in other BMP pathway components during repair. Quantitative RT-PCR analysis of total trachea (epithelium and mesenchyme) showed that, by 24 hpi, levels of transcripts for *Bmp4*, *Bmp5*, *Bmp6*, *Acvr1*, *Bmpr1a* and *Bmpr1b* were all reduced (Fig. 4A). By contrast, transcripts for the antagonist *Fst* were upregulated. Immunohistochemistry of tracheal sections from *Bmp4-nCFP* ‘knock-in' reporter mice (Fig. 4B) showed that Bmp4 is expressed at steady state predominantly in cells in the subepithelial mesenchyme, and in some luminal cells. At 24 hpi, expression is still seen in the mesenchyme, albeit at lower levels, and is absent from the epithelium. At the same time, combined *in situ* hybridization and immunohistochemistry indicated that *Fst* is upregulated in both Krt5^+^ BCs and in the mesenchyme (Fig. 4C). Our findings at 24 hpi were confirmed and extended at 48 hpi using microarray analysis of genes expressed in separated epithelial and mesenchymal cell populations (Fig. S5). For example, transcripts for *Bmp4* are reduced in both epithelium and mesenchyme at 48 hpi, whereas transcripts for *Fst* are elevated in both populations. In addition, the microarray data showed upregulation of the genes encoding the secreted BMP antagonists chordin-like 2 (Chrdl2) and follistatin-like 3 (Fstl3) in the mesenchyme, and the BMP modulator twisted gastrulation 1 (Twsg1) in the epithelium.

Taken together, these results support a model in which BMP signaling is transiently downregulated in the epithelium during repair. This reduction is likely to occur as a result of decreased expression of genes encoding BMP ligands and receptors, and the upregulation of genes encoding antagonists, in particular Fst.

### The BMP signaling inhibitor LDN regulates BC proliferation during repair *in vivo*

Given that BMP antagonists promote the proliferation of BCs and their progeny in culture and that BMP signaling through Smad1/5/8 is downregulated during repair, we asked whether giving LDN-193189 systemically after injury would enhance the regenerative process. Previous studies have used this compound to inhibit BMP signaling *in vivo* in mice (Steinbicker et al., 2011; Tsugawa et al., 2014; Yu et al., 2008), with positive effects on liver regeneration.

**Fig. 5.**
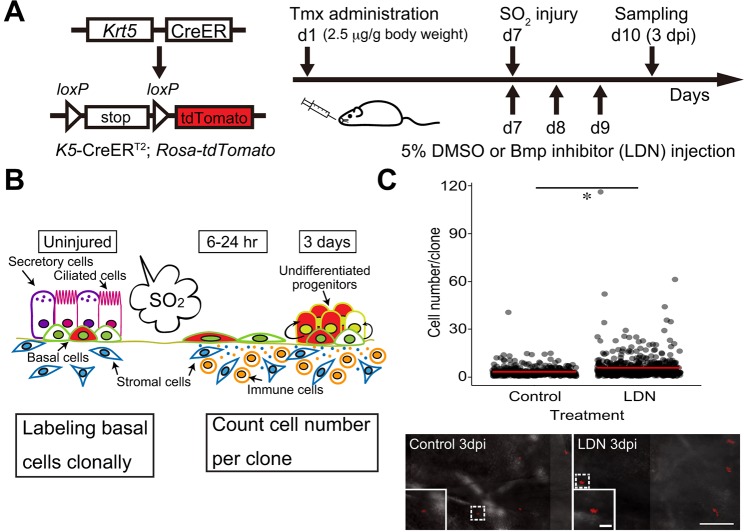
**Inhibition of BMP signaling promotes clonal expansion of BCs.** (A) Schematic of clonal analysis of BCs *in vivo*. Krt5^+^ BCs were labeled clonally with a low dose of tamoxifen (2.5 µg/g body weight). One week later, mice were given 5% DMSO or 3 mg/kg body weight LDN-193189 by i.p. injection and exposed to SO_2_ for 4 h. Mice were then treated with drug every 24 h and tracheas harvested at 3 dpi. (B) Schematic of clonal expansion of BCs after injury. Individual BCs were labeled at steady state (red) and clones expanded after injury. Both single cells and clusters were considered to be ‘clones'. (C) (Top) Clone size (cell number/clone) at 3 dpi with and without LDN-193189 treatment. Red bars show the average number of cells/clone: 3.3 for control and 5.8 for LDN-193189-treated mice, respectively. Data are from three mice. *P=5.478×10^−16^ by Mann–Whitney–Wilcoxon test. (Bottom) Whole-mount image of tracheal epithelium from *Krt5-CreER;Rosa-Tomato* mouse that had received a low dose of tamoxifen, showing typical clone distribution and size 3 days after SO_2_ injury. Inset shows higher magnification of the clone in the boxed area. Scale bar: 200 μm at low magnification and 50 μm at high magnification.

We examined the potential effect of LDN-193189 in two ways: by counting epithelial cell number in the tracheas of treated mice versus controls (see later); and by quantifying the size of clones derived from lineage-traced Krt5^+^ BCs. For clonal analysis, *Krt5-CreER;Rosa-Tomato* mice were treated with a low dose of tamoxifen (2.5 μg/g body weight) through oral gavage to induce lineage labeling of well-separated BCs (Fig. 5A). After 1 week, mice were exposed to SO_2_ for 4 h just after receiving an intraperitoneal (i.p.) injection of DMSO (control) or 3 mg/kg LDN-193189. Mice were treated with inhibitor daily and harvested at 3 days post injury (dpi) (Fig. 5B). Analysis of the tracheal epithelium showed that, on average, the number of cells per clone was small (3.3), with many cells staying as single cells, both in the dorsal and ventral trachea. However, with LDN-193189 treatment clone size was significantly increased (5.8) (Fig. 5C) in both regions. This result indicates that suppression of BMP signaling promotes BC proliferation *in vivo*.

### Evidence for active apoptotic cell extrusion during epithelial regeneration

**Fig. 6.**
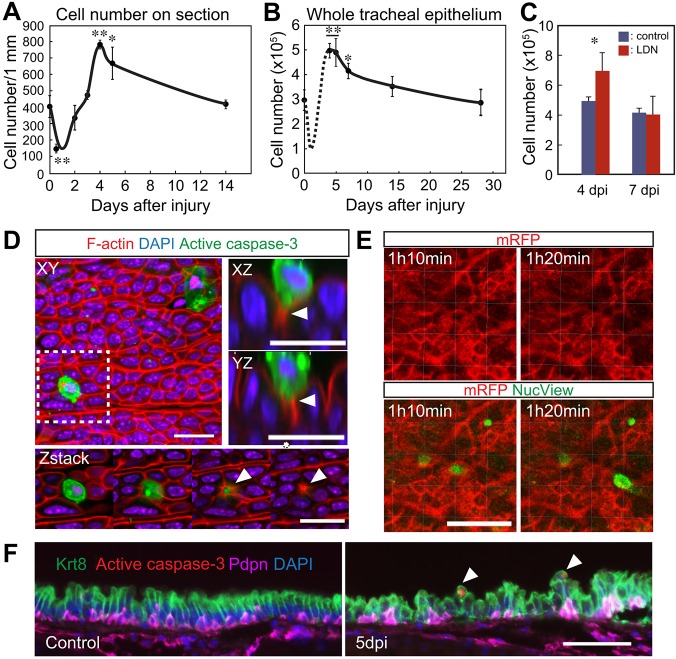
**Regulation of cell number in tracheal epithelium by cell extrusion during repair.** (A) Number of epithelial cells per mm along basal lamina after injury (*n*=3 mice). (B) Total epithelial cell number in a single trachea (*n*=3 mice). The number at day 1 is estimated from the data in A. (C) Total tracheal cell number at 4 dpi and 7 dpi with or without systemic LDN-193189 treatment (*n*=3 tracheas for control and *n*=5 tracheas for LDN-193189 treated). *P<0.05, **P<0.01 versus uninjured (*n*=3). (D) Confocal images of whole tracheal epithelium at 6 dpi after immunohistochemistry showing apoptotic cells (cleaved caspase 3^+^, green) and F-actin (red). The lower panels show images at different levels of the region boxed in the upper left panel. Upper right panels are enlarged images of an apoptotic cell being extruded. Arrowheads indicate the actin ring in neighboring cells. (E) Snapshots from live cell imaging of tracheal epithelium of a Rosa-mT/mG mouse (red marks epithelial cell membranes) exposed to the caspase 3 substrate Nucview (green). (F) Sections of trachea before injury and at 5 dpi stained with antibodies to Krt8 (luminal cells), Pdpn (BCs) and active caspase 3. The ratio of Krt8^+^ to Pdpn^+^ cells is 1.50±0.34 in the control (total cells counted=642) compared with 2.3±0.38 at 5 dpi (total cells counted=735). Arrowheads mark the site of cell extrusion. Scale bars: 20 µm in D; 30 µm in E; 50 μm in F.

Previous studies using the SO_2_ injury model demonstrated that early in repair the epithelium is more disorganized and multilayered than at steady state, or after regeneration is complete (Tadokoro et al., 2014). Quantification from histological sections (Fig. S6) shows that the number of cells per unit of basal lamina, averaged along the whole trachea, peaks at 4 dpi, at about twice the steady-state level (Fig. 6A). This correlates well with changes in the total number of epithelial cells in the trachea, a value that also peaks at ∼4-5 dpi before returning to control levels by 2 weeks (Fig. 6B). These findings suggest that after 4-5 dpi there is a dynamic loss of crowded epithelial cells to restore cell density. Mechanisms based on either apoptotic or non-apoptotic cell extrusion have been reported in other *in vivo* and *in vitro* systems involving cell crowding (Eisenhoffer et al., 2012; Macara et al., 2014). We therefore examined the surface of the regenerating tracheal epithelium using whole-mount immunohistochemistry for caspase 3, a marker for apoptotic cells. Whereas caspase 3^+^ cells are rare at steady state (Fig. 6D, Fig. S6), there are many such cells in the regenerating epithelium at 6 dpi, when cell density is declining. Moreover, individual caspase 3^+^ cells are located in the center of rosettes of columnar cells with high concentrations of apical F-actin, consistent with the squeezing out of cells by contraction of actin rings. This interpretation is supported by real-time confocal imaging of the live tracheal epithelium of Rosa membrane-targeted Tomato/membrane-targeted GFP (Rosa-mT/mG) mice treated with the fluorescent caspase substrate Nucview (Fig. 6E, Fig. S6C). Finally, immunohistochemistry of tracheal sections at 5 dpi showed that caspase 3^+^ cells are Krt8^+^ luminal cells and not Pdpn^+^ BCs (Fig. 6F). These data also clearly show that the ratio of Krt8^+^ to Pdpn^+^ cells is higher at 5 dpi than in controls (Fig. 6F, see legend).

Given these results, we examined whether LDN-193189 affects not only BC proliferation but also the accumulation and then active extrusion of luminal cells during repair. The total number of tracheal epithelial cells is increased in LDN-193189-treated mice compared with controls at 4 dpi (Fig. 6C). However, by 7 dpi there is no statistical difference in epithelial cell numbers in treated versus untreated tissue.

## DISCUSSION

Here, we use both an *in vitro* clonal organoid culture system and an *in vivo* injury model in the mouse trachea to explore mechanisms involved in the maintenance and regeneration of the pseudostratified mucociliary airway epithelium from basal progenitors. These studies are likely to be relevant to the human lung, in which the majority of the intralobar airways are lined by a pseudostratified epithelium with TRP63^+^ KRT5^+^ BCs (Rock et al., 2010). In human airways, cycles of luminal cell loss and regeneration are likely to occur as a result of infection by respiratory viruses or exposure to inhaled gases and stomach contents. Sloughing of dead epithelial cells is also reported in severe asthma (Penberthy et al., 2014). The main new finding of the current study is that regeneration of the mucociliary epithelium after loss of luminal cells involves the interplay of two counteracting processes. The first is the exuberant accumulation and multilayering of new progeny of BCs that is enabled, at least in part, by the transient downregulation of BMP signaling in the epithelium through Smad1/5/8. The second process is the active extrusion of apoptotic cells from the crowded epithelium, so that the pre-injury cell density is eventually restored.

### Dynamic expression of BMP signaling pathway components during repair

**Fig. 7.**
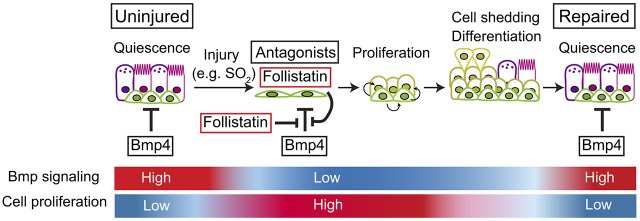
**Proposed role of BMP signaling during repair of the tracheal epithelium.** At steady state, Bmp4, expressed mainly in the mesenchyme, maintains a low rate of cell proliferation in at least a subset of the BC population. After injury, the BMP antagonist Fst is transiently upregulated both in surviving epithelium and mesenchyme. This, in turn, leads to enhanced epithelial proliferation and differentiation into luminal cells. Cell crowding leads to extrusion and shedding of apoptotic cells and ectopic BMP inhibitors do not increase the final cell density.

According to our model (Fig. 7), BMP signaling in the pseudostratified mucociliary airway epithelium normally acts as a brake on cell proliferation and helps to keep BCs quiescent. A critical event in regeneration after loss of luminal cells by SO_2_ injury is the downregulation of BMP signaling, manifest as a decrease in epithelial phospho-Smad1/5/8 levels (Fig. 3). Taken together, our evidence from both *in vitro* and *in vivo* approaches suggests that this decrease in BMP signaling involves several interrelated processes. These include a reduced expression of BMP ligands – for example Bmp4 in the mesenchyme – as well as decreased expression of receptors and increased levels of BMP antagonists in both epithelium and mesenchyme.

The model that we propose is not without complications that need to be resolved by further studies. For example, the gene encoding Bmp5 shows higher expression in the trachea than Bmp4, yet the protein apparently has no effect on the tracheosphere assay (Fig. S2). Differential effects of BMP subclasses through different receptors have been reported in other systems (Lavery et al., 2008). It is also possible that Bmp5 normally functions in the airway as a heterodimer, with Bmp2 for example. Another complication is that, whereas transcripts for *Fst* increase significantly after injury, transcripts for other antagonists are decreased (e.g. *Twsg1*, *Bambi* and *Crim1* in the mesenchyme, and *Chrd* in the total trachea) (Fig. 4, Fig. S5). This discrepancy might reflect heterogeneity of cell types in these populations, especially the mesenchyme, and future clarification might come from the analysis of gene expression changes in specific subpopulations of single cells. Certainly, Fst shows the largest change in expression during repair (2.4-fold increase in the mesenchyme and 30-fold increase in the epithelium at 48 hpi). However, this antagonist can bind both BMPs and activins, so it is possible that part of its function is to block signaling through non-BMP pathways *in vivo*. Currently, we do not know why exogenous Fst has only a small effect on cell proliferation in the tracheosphere assay, whereas Chrd, LDN-193189 and DMH1/2 efficiently promoted proliferation (Fig. 2). In an intestinal organoid culture assay system the effect of the BMP antagonist Chrdl2 was only seen under certain culture conditions and not others (Seiler et al., 2015). Finally, we do not know which cells in the tracheal mesenchyme express Fst, Fstl3 and Chrdl2 and whether signals from inflammatory cells induce upregulation, as seen in other systems (Akiyama et al., 2013).

There is ample and compelling evidence from studies of other epithelial tissues, such as skin and hair follicles and mammalian intestine, that BMP signaling through phospho-Smad1/5/8 functions as a negative regulator of stem/progenitor cell proliferation. Moreover, expression of antagonists plays an important role in orchestrating repair and regeneration (Genander et al., 2014; Hsu et al., 2014; Kandyba et al., 2013; Kosinski et al., 2007; Lewis et al., 2014; Oshimori and Fuchs, 2012; Seiler et al., 2015). In the case of the hair follicle there is evidence that BMP signaling not only regulates progenitor cell proliferation but also cell fate choice in differentiation. However, in our studies the current data do not support such a role, and it appears that the effect of BMP signaling in the large airways of the adult lung is largely through proliferation.

### Cell shedding as part of a rheostat controlling airway epithelial architecture

Previous lung studies had shown that apoptotic airway epithelial cells can be phagocytosed by other epithelial cells in a Rac1-dependent manner, at least in the context of asthma (Juncadella et al., 2013; Penberthy et al., 2014). Here, we present the first evidence, using live cell imaging in the context of airway regeneration after injury, that apoptotic cells are squeezed out of the crowded epithelium by the constriction of neighbors. As a result, the epithelial crowding seen in the first few days after injury is relieved and normal density is attained. Moreover, it appears that compounds such as LDN-193189 that promote epithelial proliferation can enhance repair by transiently increasing cell density during early phases of regeneration, but do not change the final tissue composition. These findings raise interesting questions about the mechanisms that initiate and terminate cell shedding during repair, and what determines final epithelial cell density and packing at different levels along the conducting airways. In many tissues a crucial role has been identified for the Hippo-Yap pathway in linking properties such as cell polarity and shape, mechanical tension, matrix stiffness and packing density to epithelial cell proliferation (Gumbiner and Kim, 2014; Macara et al., 2014). Significantly, recent studies have shown that loss of Yap from tracheal BCs leads to conversion of the pseudostratified tracheal epithelium into a simple columnar epithelium, while overexpression leads to hyperplasia and stratification rather than cell shedding (Zhao et al., 2014). Precisely how Yap signaling changes during regeneration of the mucociliary epithelium and how this interacts with the BMP signaling pathway and cell shedding mechanisms remain to be determined.

## MATERIALS AND METHODS

### Animals

*Krt5*-CreER^T2^ (Van Keymeulen et al., 2011), *Rosa-tdTomato* (Rock et al., 2011a), *Foxj1-GFP* (Ostrowski et al., 2003), *Rosa-mT/mG* and *Pdfgra^tm11(EGFP)Sor^* (The Jackson Laboratory) were maintained on a C57BL/6 background. *Bmp4-nCFP* (Jang et al., 2010) was maintained on an ICR background. All experiments were performed in accordance with IACUC-approved protocols.

### Tracheosphere culture

Ngfr^+^ BCs isolated as described (Rock et al., 2009) from C57BL/6 or *Foxj1-GFP* mice were suspended in MTEC/plus medium (Rock et al., 2009), mixed 1:1 with growth factor-reduced Matrigel (Corning Life Sciences), and seeded at 1000 cells/well in 24-well 0.4 μm pore inserts (#3470, Corning Life Sciences). Factors were added to the medium in the lower well, and the medium changed every other day. MTEC/SF medium (Rock et al., 2009) was used from day 7. Images were taken using an AxioVert 200M (Carl Zeiss). Spheres were counted at day 9 and dissociated using dispase (BD Biosciences, 354235; 70 µl/well at 37°C for 30 min) and 0.1% trypsin/EDTA (GIBCO, 15400-054) and cell number counted using a hemocytometer. For quantifying GFP^+^ cells, dissociated cells were fixed with 2% paraformaldehyde (PFA) in PBS, and analyzed using FACSCanto (BD Biosciences). For quantifying proliferation, spheres were incubated in 10 μM EdU for 2 h and staining carried out using Click-iT EdU Imaging Kit (Invitrogen). Bmp4, Bmp5, recombinant mouse Chrd and follistatin 288 were from R&D Systems. LDN-193189 was from Stemgent, recombinant mouse Nog was from PeproTech and DMH1 was from Sigma (D8946).

### Immunohistochemistry

Mouse tracheas were fixed with 4% PFA in PBS at 4°C for 4 h, washed with PBS, and embedded in paraffin for sectioning. Tracheas were sectioned longitudinally in the midline along the dorsal-ventral axis at 7 µm. Sections were deparaffinized, rehydrated and subjected to antigen retrieval in 10 mM sodium citrate (pH 6.0) at 121°C for 10 min. After blocking with 10% donkey serum, 3% BSA and 0.1% Triton X-100 in PBS, sections were incubated with primary antibodies in blocking buffer at 4°C overnight. For immunohistochemistry of phospho-Smad, optimal results were obtained if sections were subsequently incubated at 37°C for 2 h. Primary antibodies were: rabbit Krt5 (1:1000; Covance, PRB-160P); mouse Trp63 (1:100; Santa Cruz, SC-8431); rabbit phospho-Smad1/5/8 (1:500; gift from Dr Edward Laufer, Columbia University); chicken GFP (1:500; Aves Labs, GFP1020; this antibody reacts with both GFP and CFP proteins); rabbit active caspase 3 (1:200; BD Biosciences, 559565); mouse acetylated tubulin (1:1000; Sigma, T7451); and rabbit Scgb3a2 (1:500; gift from Dr Shioko Kimura, National Cancer Institute NIH Bethesda). Alexa Fluor-labeled secondary antibodies (Invitrogen and Jackson ImmunoResearch) were used at 1:500 dilution. For detecting F-actin, samples were incubated with Alexa Fluor 555-labeled phalloidin (1:40). After secondary antibody staining, nuclei were stained with DAPI, and sections mounted in FluoSaver (Calbiochem). Confocal images were obtained using an LSM 710 inverted confocal microscope (Carl Zeiss).

### *In situ* hybridization

Paraffin sections were deparaffinized and rehydrated, and treated with proteinase K (20 µg/ml, Invitrogen) for 10 min followed by acetylation with triethanolamine for 10 min at room temperature. After prehybridization, DIG-labeled probes (300 ng/ml) were hybridized at 65°C overnight. For the *Fst in situ* probe see Table S2. After washing once with 2×SSC for 20 min and four times for 20 min each with 0.2×SSC at 65°C, slides were blocked with 10% heat-inactivated sheep serum in TBS (50 mM Tris-HCl, 50 mM NaCl pH 7.5) for 1 h, and incubated with HRP-conjugated sheep anti-DIG antibody (1:1000; Roche Applied Science, 11207741910) in 1% heat-inactivated sheep serum/PBS at 4°C overnight. To detect Krt5, slides were incubated with anti-Krt5 antibody followed by secondary antibody and DAPI for counterstaining. Slides were incubated with TSA-Cy3 (PerkinElmer) for 10 min.

### Quantitative RT-PCR and western blot analysis

Total RNA was extracted from whole tracheas using the Direct-zol RNA MiniPrep Kit (Zymo Research). cDNA was synthesized using the iScript cDNA Synthesis Kit (Bio-Rad), and quantitative RT-PCR was performed with iQ SYBR Green Supermix (Bio-Rad) using a StepOne Plus system (Applied Biosystems). For primer sequences see Table S2.

Western blot analysis was performed on protein extracts from total trachea, epithelium and mesenchyme. Equal amounts of protein were separated by SDS-PAGE and transferred onto polyvinylidene fluoride membranes. Membranes were blocked for 1 h with 5% (w/v) dried milk in PBS containing 0.1% Tween 20, and incubated with phospho-Smad1/5/8 antibody (1:1000; Cell Signaling, 9511), phospho-p38 antibody (1:2000; Cell Signaling, 4511), phospho-SAPK/JNK (Thr183/Tyr185) (G9) antibody (1:1000; Cell Signaling, 9255) and β-actin antibody (1:3000; Abcam, ab8226) in blocking buffer overnight at 4°C, followed by HRP-conjugated secondary antibody (Bio-Rad). Proteins were visualized using the ECL detection system (FEMTOMAX-110, Rockland Immunochemicals). The phospho-Smad1/5/8 band was validated by a positive control (HMEC1 cell treated with Bmp9) and phospho-p38 and phospho-Jun bands by molecular weight.

### Microarray analysis

Epithelium isolated by protease digestion from control and 48 hpi tracheas was incubated in 0.1% trypsin, 1.6 mM EDTA for 20 min at 37°C, followed by gentle pipetting. Mouse CD45 (Ptprc) MicroBeads (Miltenyi Biotec) were used to deplete CD45^+^ leukocytes. The mesenchyme remaining after removal of epithelium was frozen in liquid nitrogen and ground up before RNA extraction. Four tracheas were used per biological replicate.

RNA was extracted using the RNeasy Micro Kit (Qiagen) and quality checked with a 2100 Bioanalyzer (Agilent Technologies). RNA was processed using Ambion MessageAmp Premier by the Duke Microarray Facility. Standard Affymetrix protocols and GeneChip Mouse Genome 430 2.0 were used to generate .cel files. Genomics Suite 6.5 (Partek) was used to perform data analysis. Robust multi-chip analysis (RMA) normalization was performed on each data set. Two-way ANOVA and fold change analyses were performed to select target genes differentially expressed between control and 48 hpi data sets of both epithelium and mesenchyme. The top differentially expressed genes in the BMP signaling pathway were selected with *P*<0.05 based on ANOVA test. Data have been deposited at NCBI GEO with accession number GSE69058.

### SO_2_ injury and repair model

Male mice (8-12 weeks age) were exposed to 500 ppm SO_2_ for 4 h. In some experiments mice were injected with 5% DMSO (v/v) or 500 µM LDN-193189 (4-{6-[4-(piperazin-1-yl)phenyl]pyrazolo[1,5-a]pyrimidin-3-yl}quinoline) to give a dose of 3 mg/kg body weight through i.p. injection just before exposure and at 24 h intervals thereafter.

### Quantification of cell numbers in tracheal epithelium during repair

Images were taken at three different positions (two ventral and one dorsal) along midline longitudinal sections of tracheas (*n*=3 for each time). For quantification of whole epithelium, tracheas from the larynx to just above the carina were incubated with dispase, the epithelium peeled from the basement membrane, and cells counted after trypsinization. Control experiments using flow cytometry showed that 98.3% of the cells are Epcam^+^ at 4 dpi.

### *In vivo* clonal analysis

Male mice at 8-12 weeks of age were given tamoxifen in corn oil (2.5 µg/g body weight) through oral gavage. One week later, mice were exposed to SO_2_ with or without LDN-193189 treatment and tracheas harvested at 3 dpi. Tiled images of whole trachea were obtained from three control and three LDN-193189-treated mice using an LSM 710 inverted confocal microscope (Carl Zeiss). Clones separated by at least five cell lengths were counted. A total of 384 and 553 clones were counted from control and LDN-193189-treated tracheas, respectively. Statistical significance was determined by Mann–Whitney–Wilcoxon test.

### Live cell imaging

Tracheas from Rosa-mT/mG mice 6 days after SO_2_ injury were harvested with minimal distortion. They were opened longitudinally and incubated at 37°C in Hanks' balanced salt solution with added Ca^2+^ and Mg^2+^, 20 mM HEPES (pH 7.4), 10 mM glucose and Nucview 488 caspase 3 substrate (Biotium, 1 μM final concentration) added 15 min before imaging. The tracheas were held flat with a slice anchor (Warner Instruments, #64-0266) and time-lapse images taken every 10 min for up to 2 h using a water-immersion lens and a Leica SP8 confocal microscope.

### Statistical analysis

All results are mean±s.d. For sphere assays (except the initial screen, which was performed in duplicate) triplicate wells were set up using cells pooled from multiple tracheas. Statistical significance was determined by unpaired Student's *t*-tests unless stated otherwise.
